# Supplementary figures and images for: Topological stress triggers persistent DNA lesions in ribosomal DNA with ensuing formation of PML-nucleolar compartment
Source: eLife. 2024 Oct 10;12:RP91304. doi: 10.7554/eLife.91304 (PMC11466457; doi:10.7554/eLife.91304)

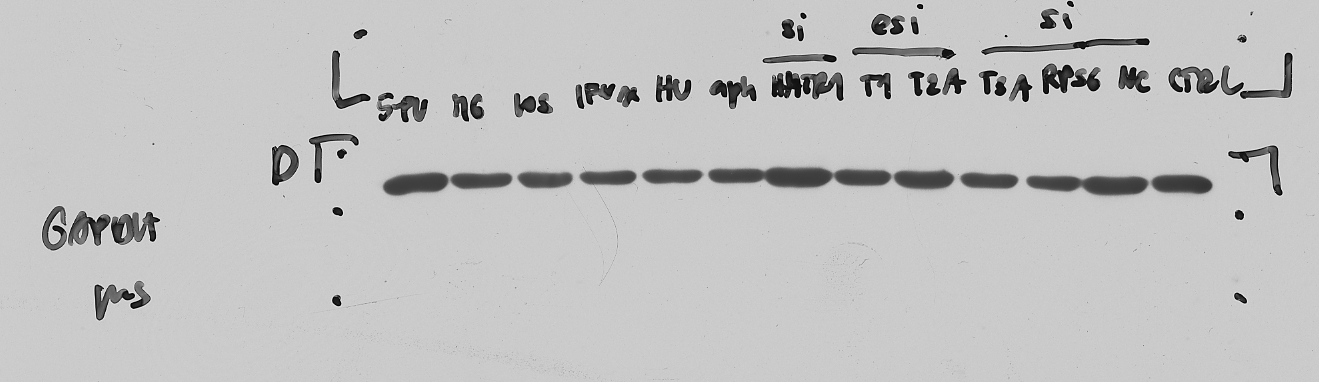

Supplement: Figure 1—figure supplement 3—source data 1. [file elife-91304-fig1-figsupp3-data1.zip › Figure 1_Figure supplement 3_Source data 1_RAW membranes/F1-FS3 ABC_GAPDH_5-FU....tif]

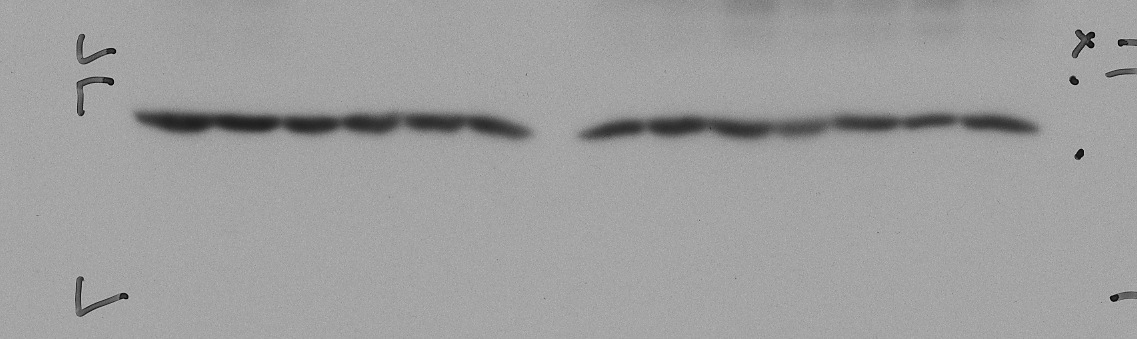

Supplement: Figure 1—figure supplement 3—source data 1. [file elife-91304-fig1-figsupp3-data1.zip › Figure 1_Figure supplement 3_Source data 1_RAW membranes/F1-FS3 ABC_GAPDH_acla.tif]

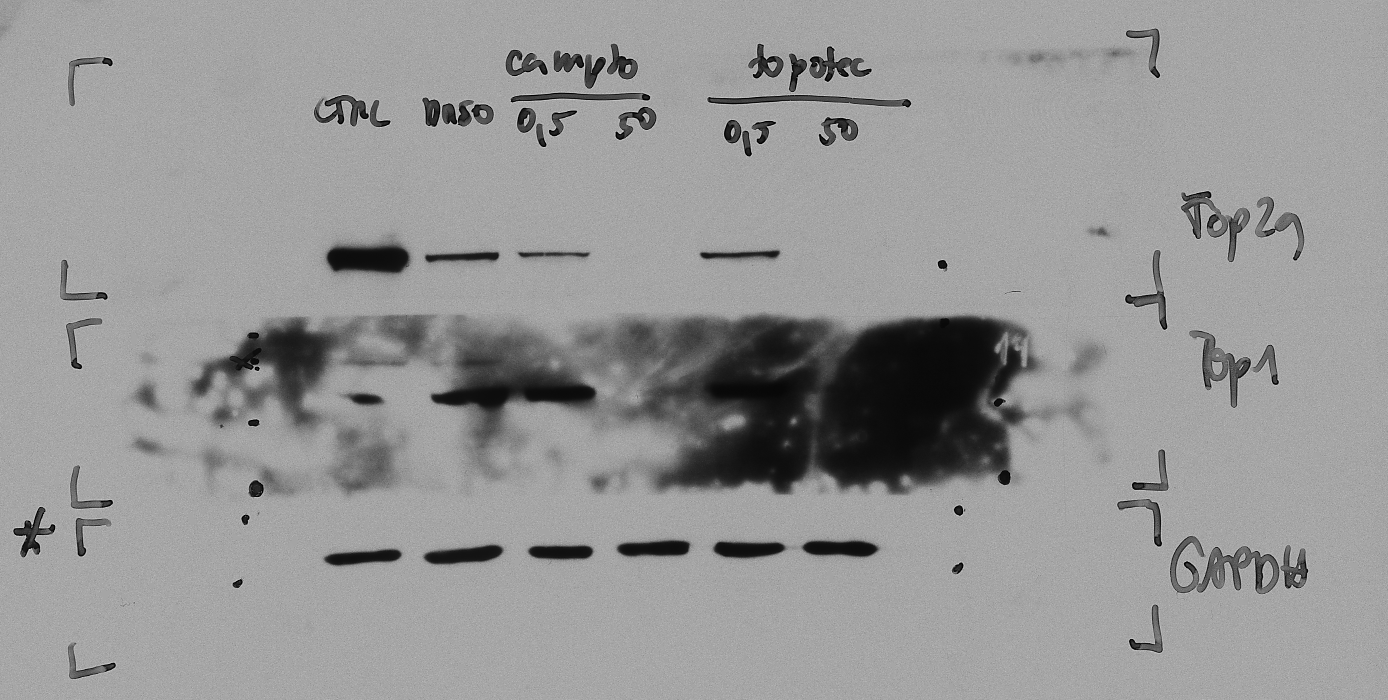

Supplement: Figure 1—figure supplement 3—source data 1. [file elife-91304-fig1-figsupp3-data1.zip › Figure 1_Figure supplement 3_Source data 1_RAW membranes/F1-FS3 ABC_GAPDH_CPT....tif]

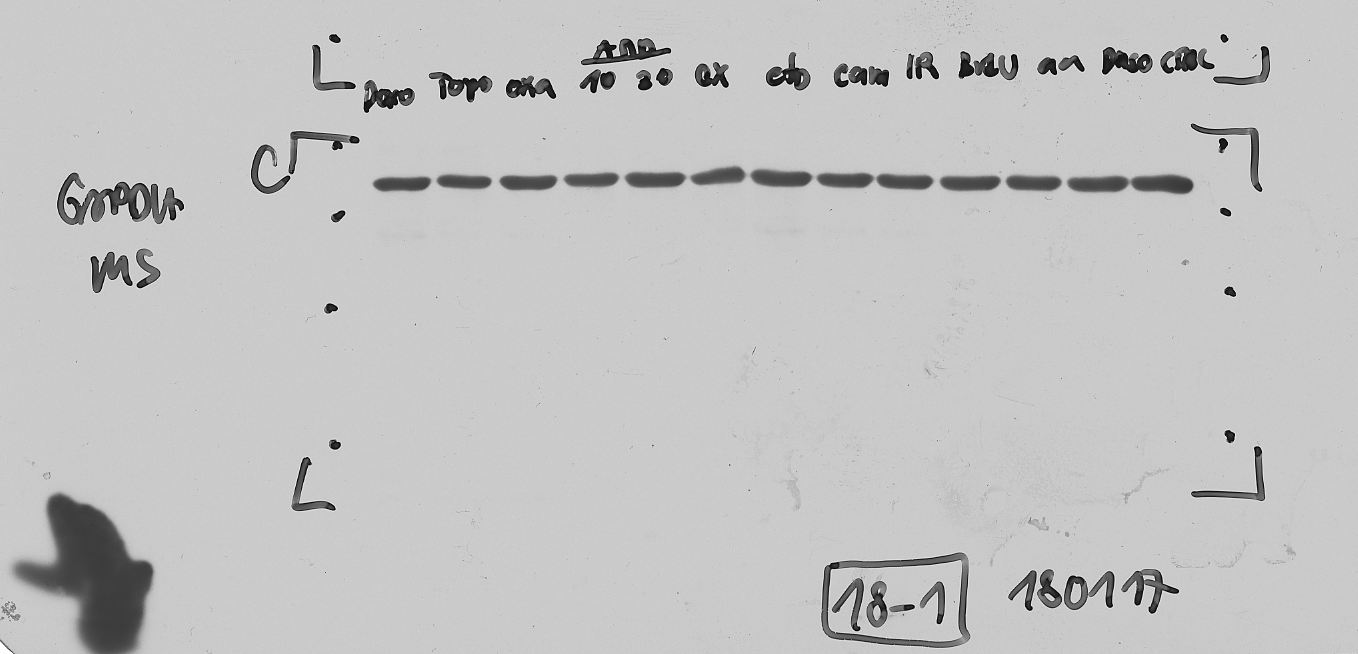

Supplement: Figure 1—figure supplement 3—source data 1. [file elife-91304-fig1-figsupp3-data1.zip › Figure 1_Figure supplement 3_Source data 1_RAW membranes/F1-FS3 ABC_GAPDH_doxo....tif]

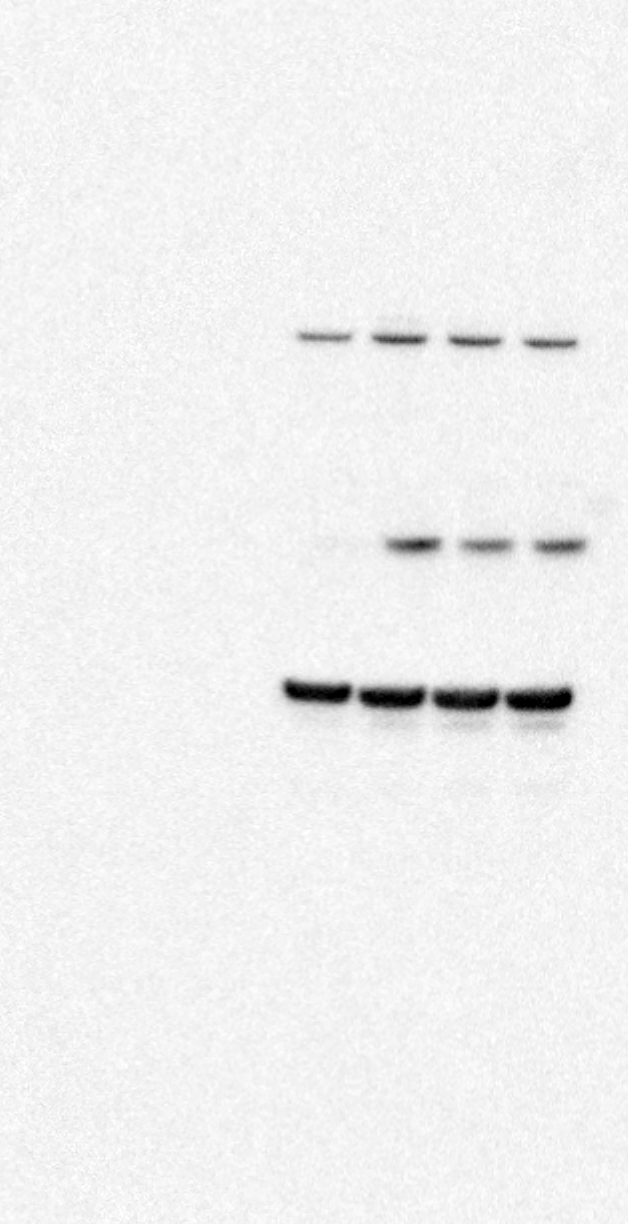

Supplement: Figure 1—figure supplement 3—source data 1. [file elife-91304-fig1-figsupp3-data1.zip › Figure 1_Figure supplement 3_Source data 1_RAW membranes/F1-FS3 AC_p53_TOP1_GAPDH_BMH21.tif]

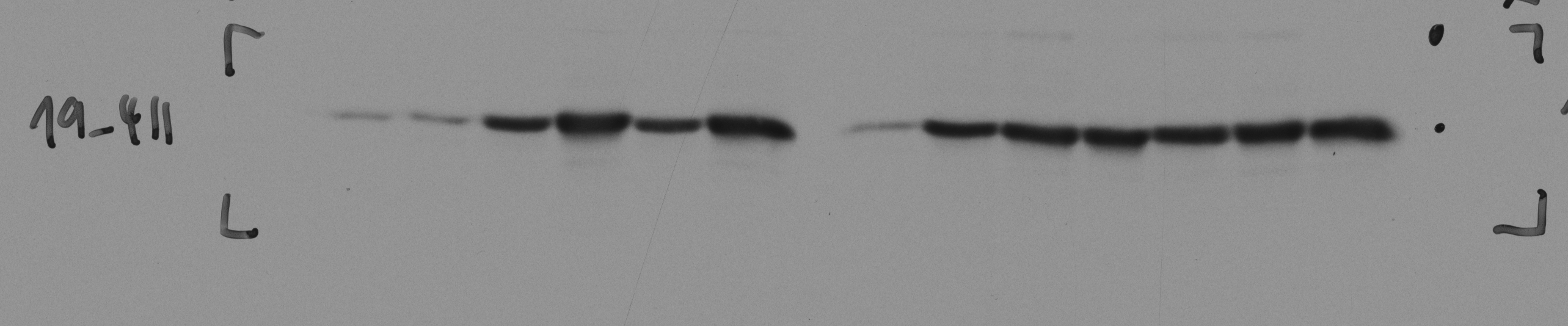

Supplement: Figure 1—figure supplement 3—source data 1. [file elife-91304-fig1-figsupp3-data1.zip › Figure 1_Figure supplement 3_Source data 1_RAW membranes/F1-FS3 A_p53p53_acla.tif]

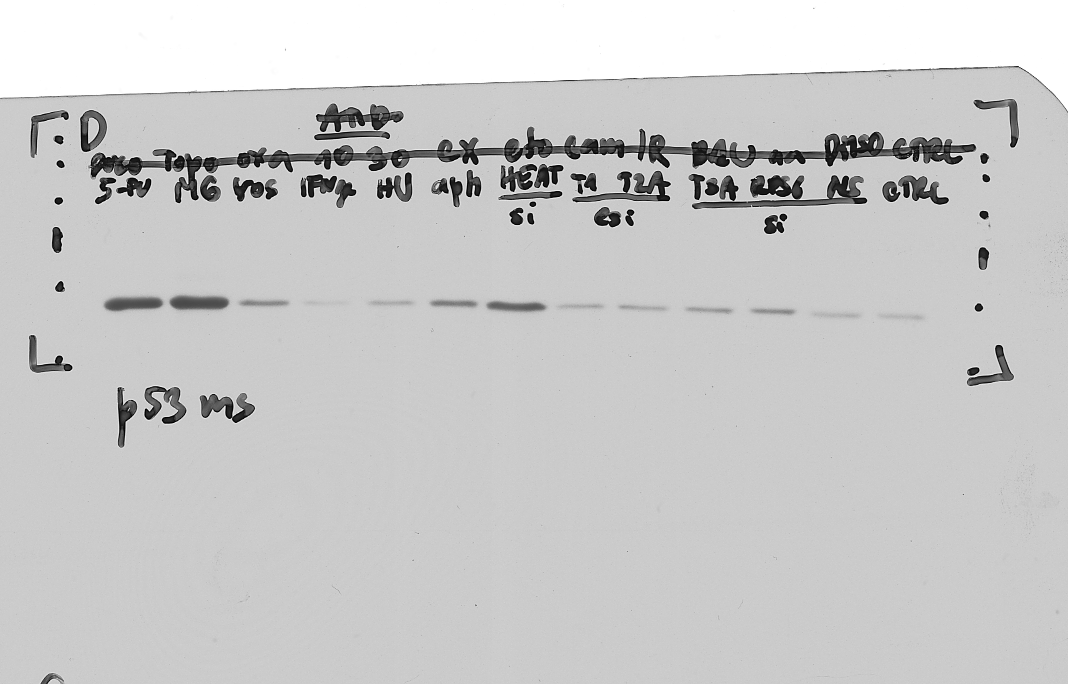

Supplement: Figure 1—figure supplement 3—source data 1. [file elife-91304-fig1-figsupp3-data1.zip › Figure 1_Figure supplement 3_Source data 1_RAW membranes/F1-FS3 A_p53_5-FU....tif]

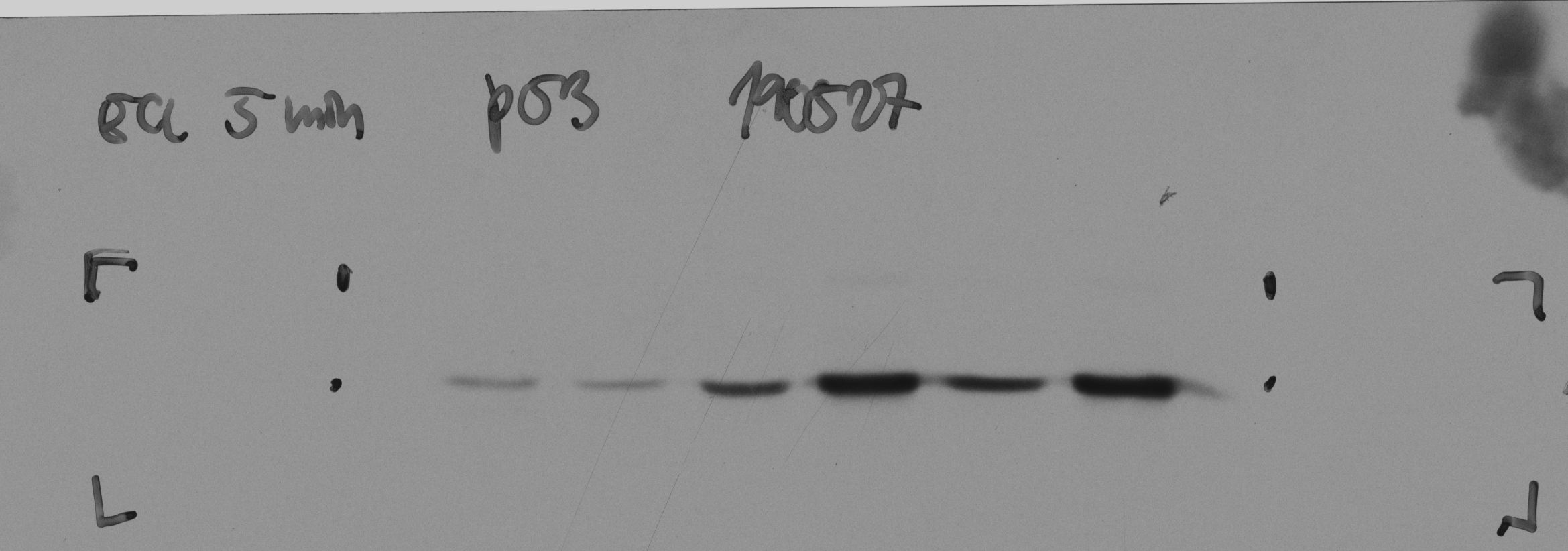

Supplement: Figure 1—figure supplement 3—source data 1. [file elife-91304-fig1-figsupp3-data1.zip › Figure 1_Figure supplement 3_Source data 1_RAW membranes/F1-FS3 A_p53_CPT....tif]

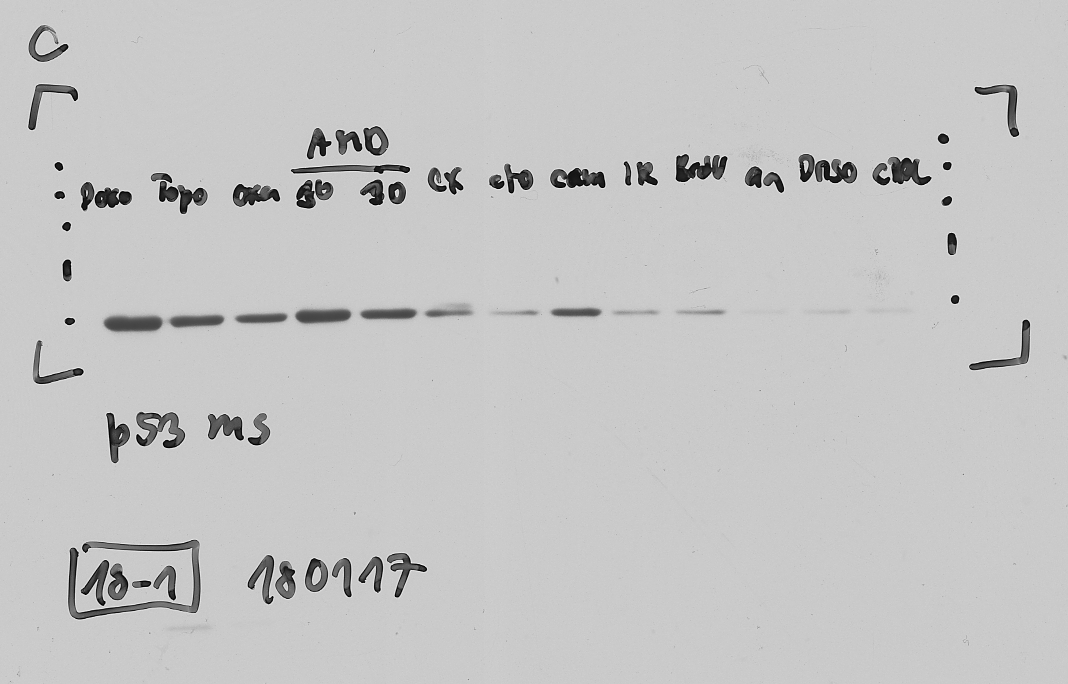

Supplement: Figure 1—figure supplement 3—source data 1. [file elife-91304-fig1-figsupp3-data1.zip › Figure 1_Figure supplement 3_Source data 1_RAW membranes/F1-FS3 A_p53_doxo....tif]

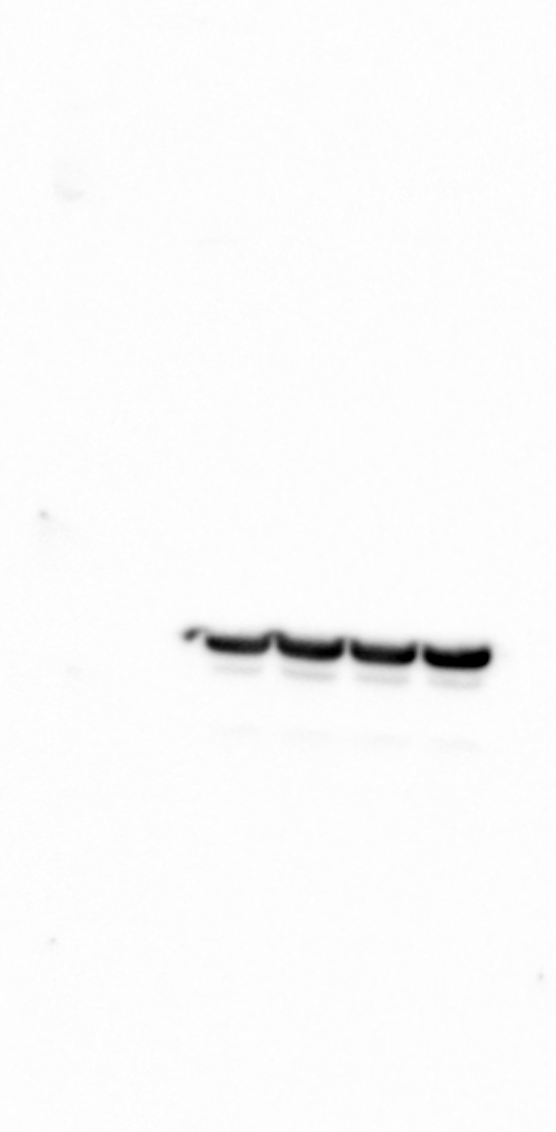

Supplement: Figure 1—figure supplement 3—source data 1. [file elife-91304-fig1-figsupp3-data1.zip › Figure 1_Figure supplement 3_Source data 1_RAW membranes/F1-FS3 B_GAPDH_BMH21.tif]

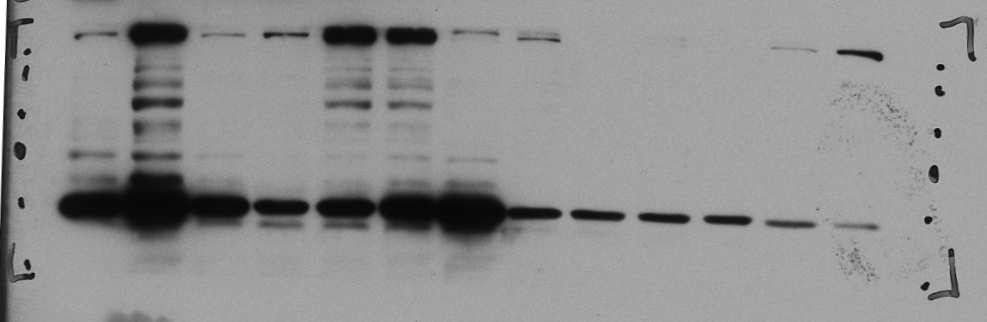

Supplement: Figure 1—figure supplement 3—source data 1. [file elife-91304-fig1-figsupp3-data1.zip › Figure 1_Figure supplement 3_Source data 1_RAW membranes/F1-FS3 B_TOP2A_5-FU....tif]

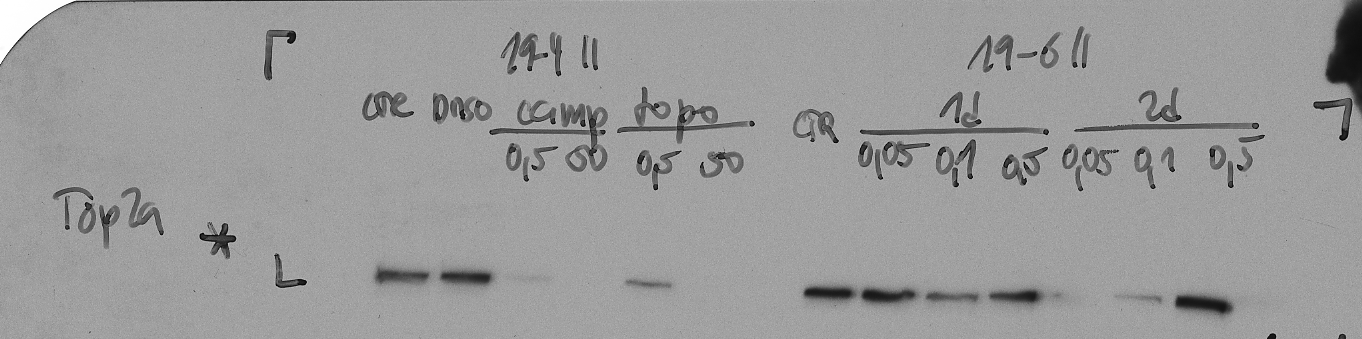

Supplement: Figure 1—figure supplement 3—source data 1. [file elife-91304-fig1-figsupp3-data1.zip › Figure 1_Figure supplement 3_Source data 1_RAW membranes/F1-FS3 B_TOP2A_acla.tif]

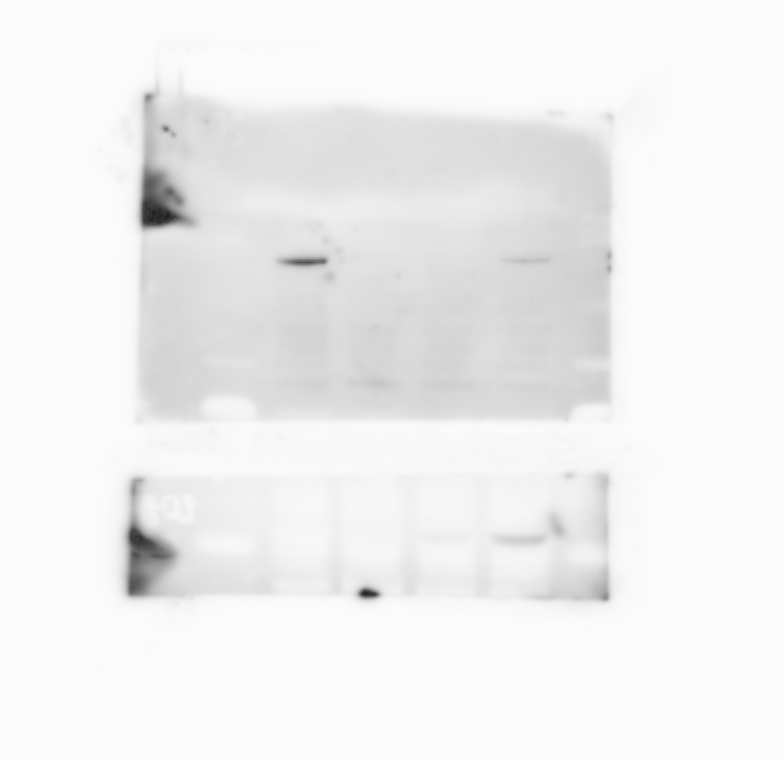

Supplement: Figure 1—figure supplement 3—source data 1. [file elife-91304-fig1-figsupp3-data1.zip › Figure 1_Figure supplement 3_Source data 1_RAW membranes/F1-FS3 B_TOP2A_BMH21.tif]

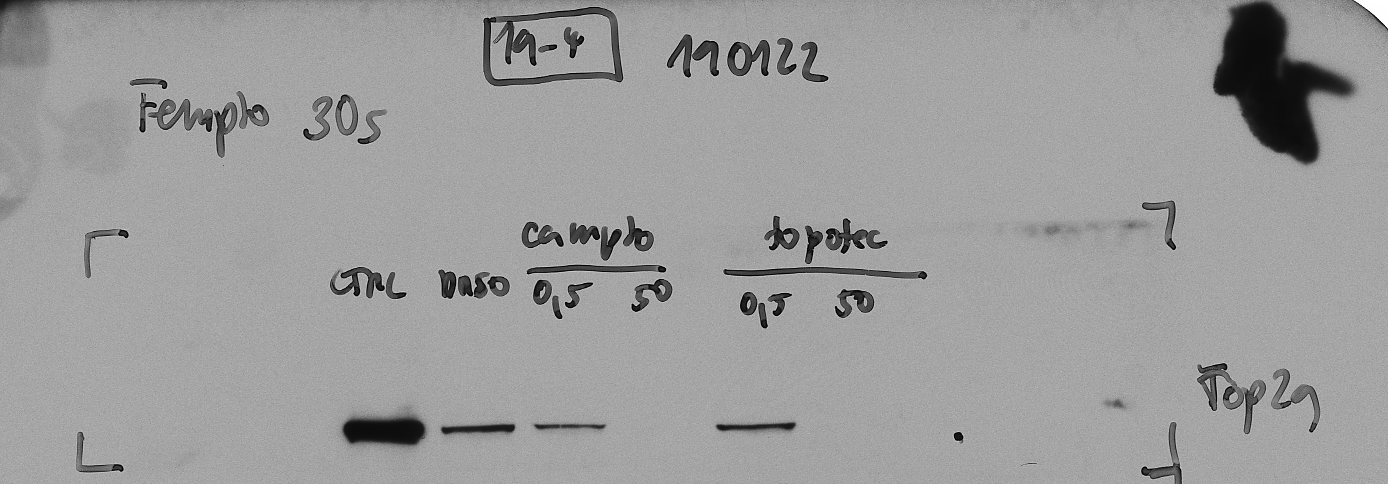

Supplement: Figure 1—figure supplement 3—source data 1. [file elife-91304-fig1-figsupp3-data1.zip › Figure 1_Figure supplement 3_Source data 1_RAW membranes/F1-FS3 B_TOP2A_CPT....tif]

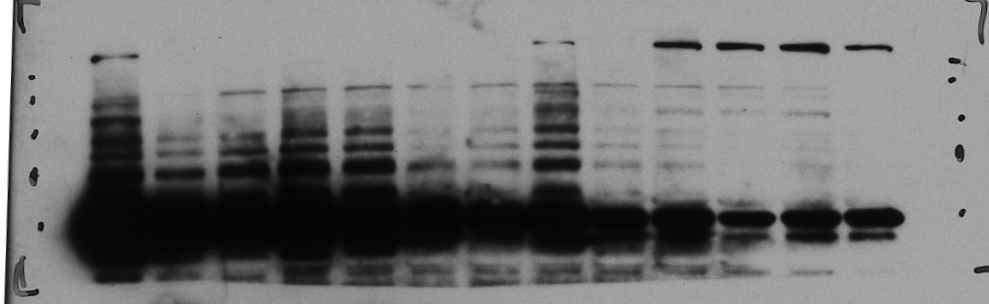

Supplement: Figure 1—figure supplement 3—source data 1. [file elife-91304-fig1-figsupp3-data1.zip › Figure 1_Figure supplement 3_Source data 1_RAW membranes/F1-FS3 B_TOP2A_doxo....tif]

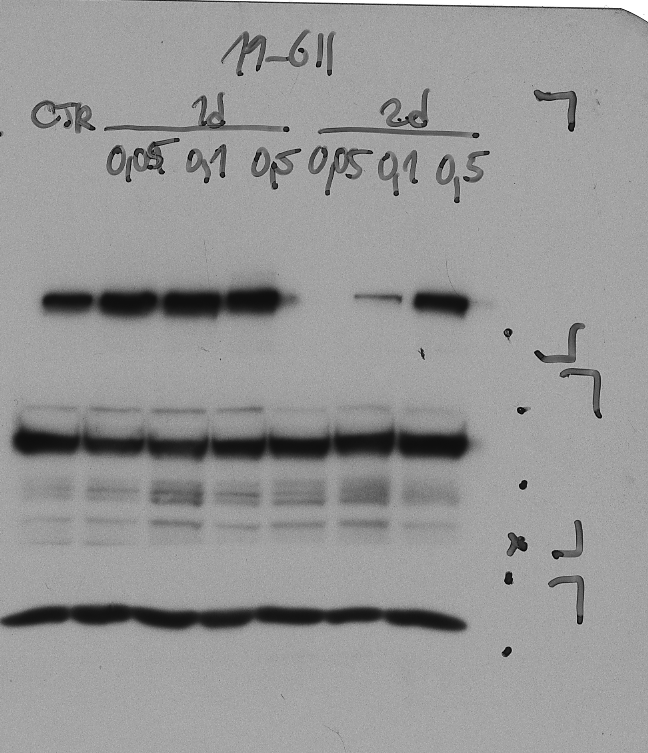

Supplement: Figure 1—figure supplement 3—source data 1. [file elife-91304-fig1-figsupp3-data1.zip › Figure 1_Figure supplement 3_Source data 1_RAW membranes/F1-FS3 C_TOP1-GAPDH_acla....tif]

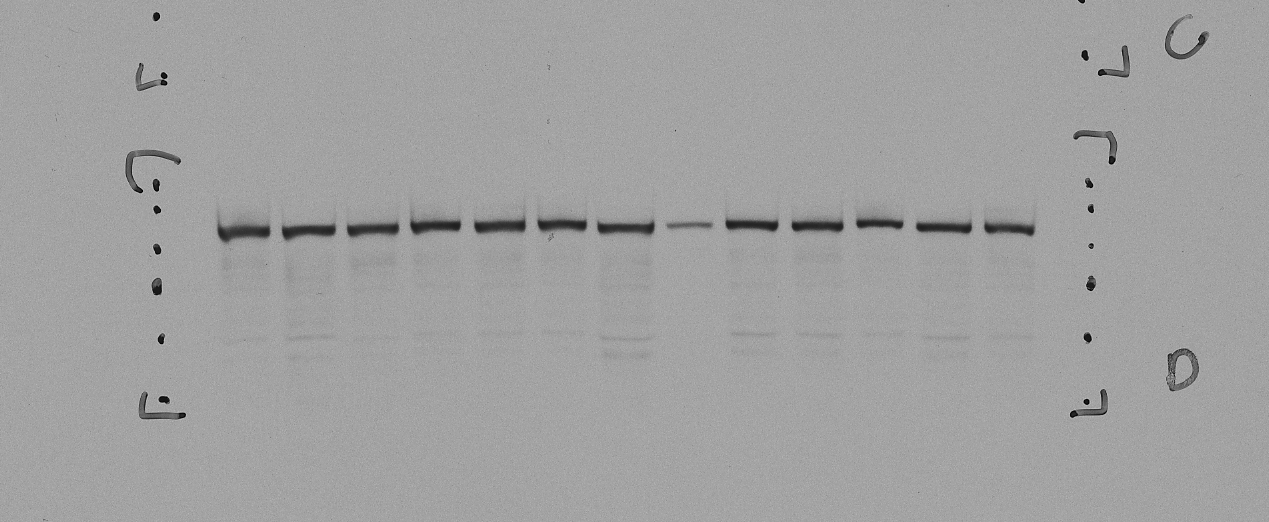

Supplement: Figure 1—figure supplement 3—source data 1. [file elife-91304-fig1-figsupp3-data1.zip › Figure 1_Figure supplement 3_Source data 1_RAW membranes/F1-FS3 C_TOP1_5-FU....tif]

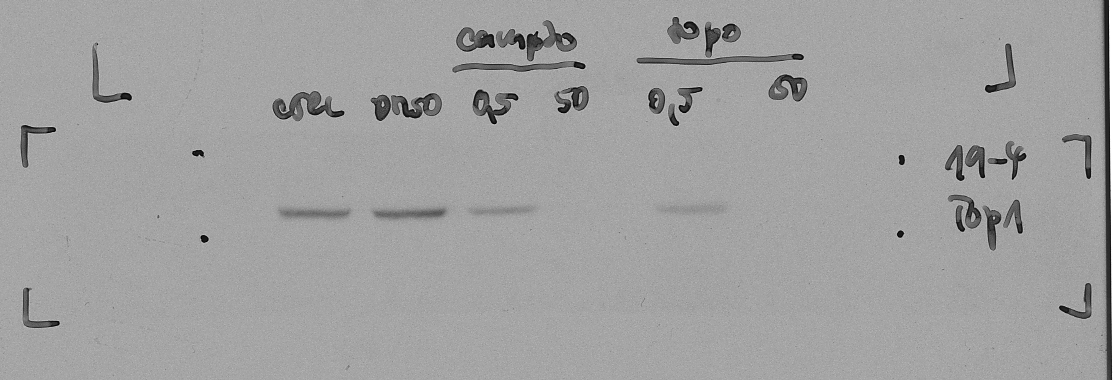

Supplement: Figure 1—figure supplement 3—source data 1. [file elife-91304-fig1-figsupp3-data1.zip › Figure 1_Figure supplement 3_Source data 1_RAW membranes/F1-FS3 C_TOP1_CPT0.5-50_TPT0.5-50_.tif]

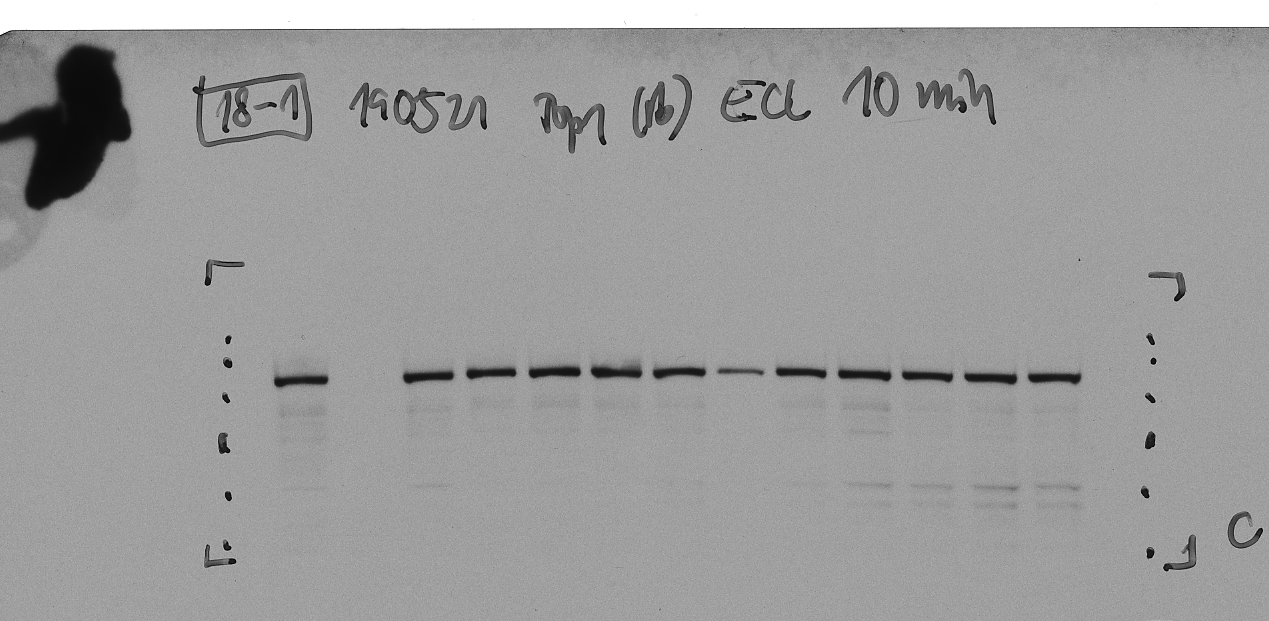

Supplement: Figure 1—figure supplement 3—source data 1. [file elife-91304-fig1-figsupp3-data1.zip › Figure 1_Figure supplement 3_Source data 1_RAW membranes/F1-FS3 C_TOP1_doxo....tif]

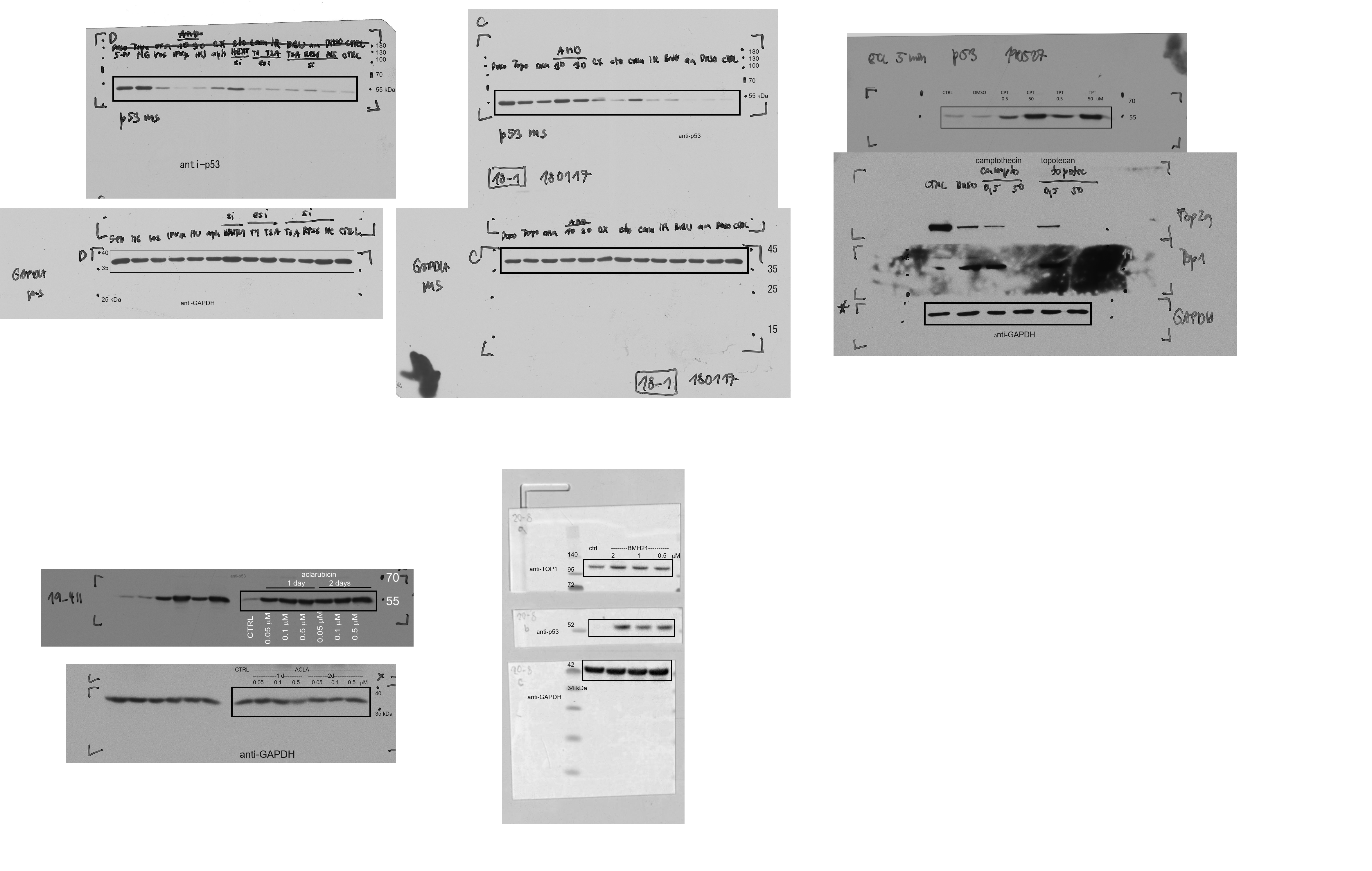

Supplement: Figure 1—figure supplement 3—source data 2. [file elife-91304-fig1-figsupp3-data2.zip › Figure 1_Figure supplement 3_Source data 2_uncropp label/Fig1_FS3-A.tif]

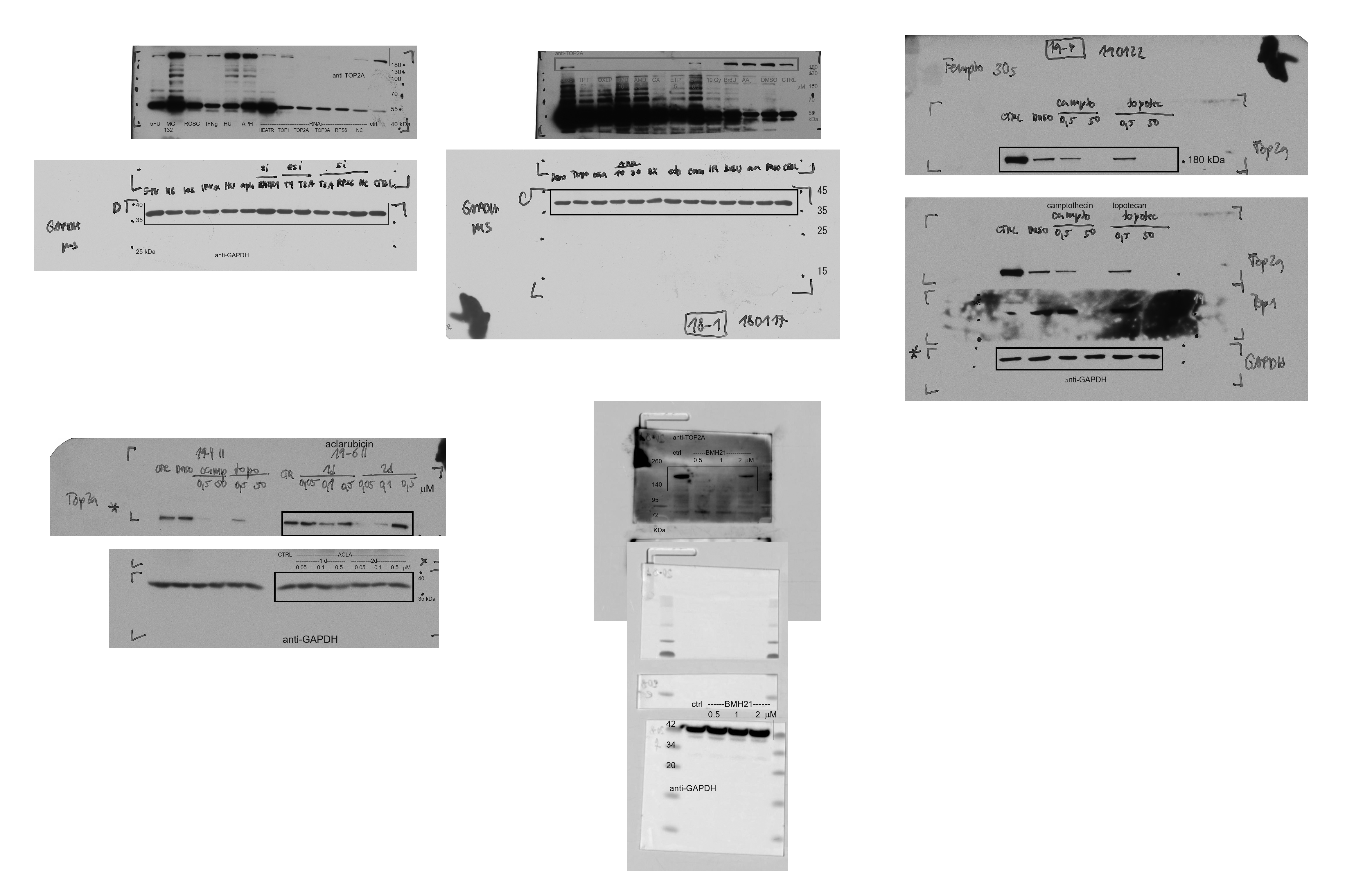

Supplement: Figure 1—figure supplement 3—source data 2. [file elife-91304-fig1-figsupp3-data2.zip › Figure 1_Figure supplement 3_Source data 2_uncropp label/Fig1_FS3-B.tif]

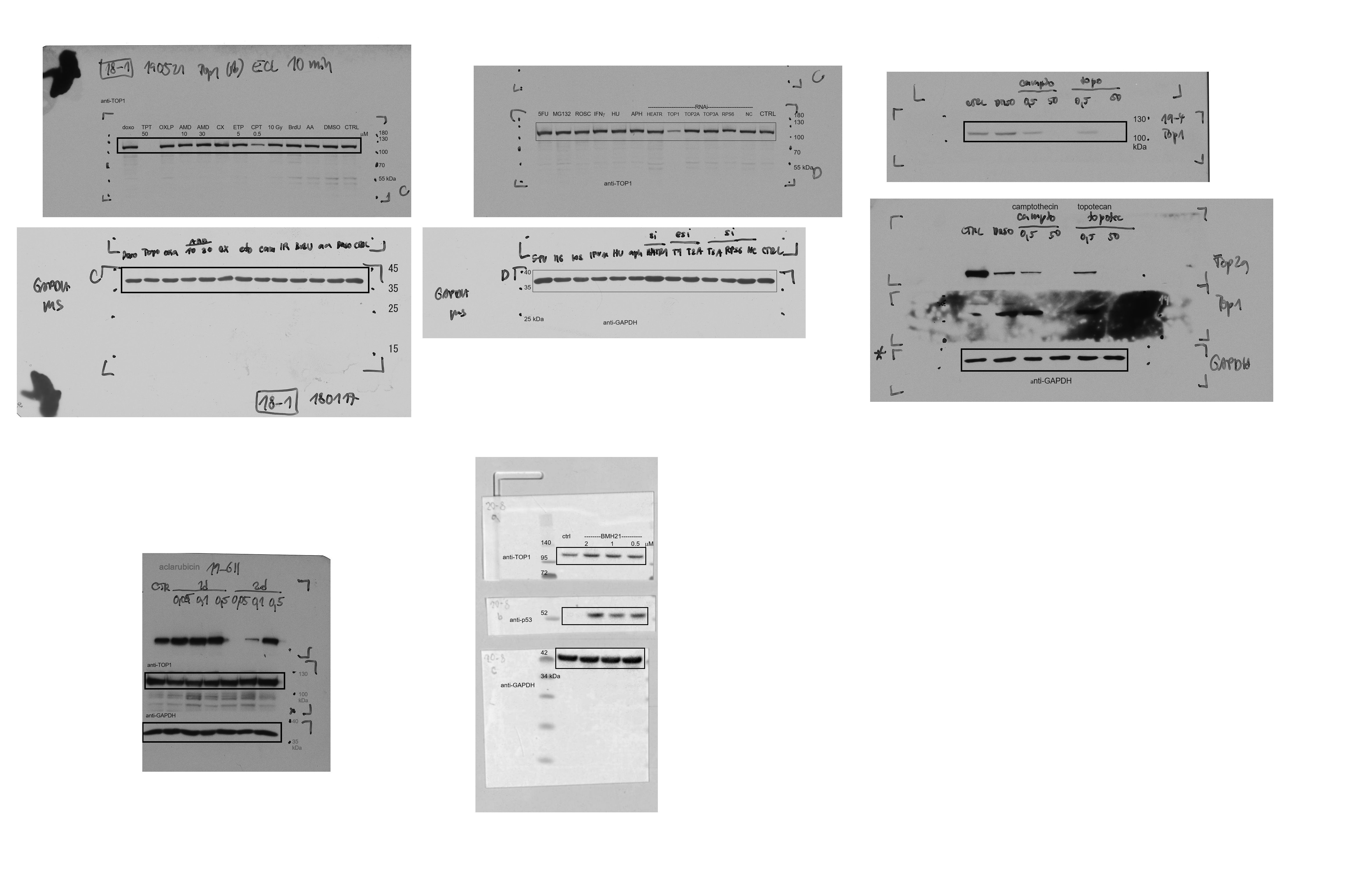

Supplement: Figure 1—figure supplement 3—source data 2. [file elife-91304-fig1-figsupp3-data2.zip › Figure 1_Figure supplement 3_Source data 2_uncropp label/Fig1_FS3-C.tif]

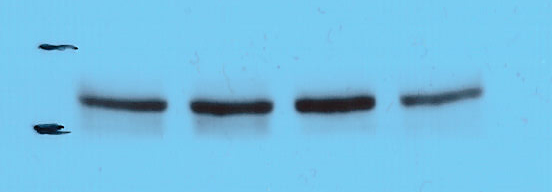

Supplement: Figure 1—figure supplement 4—source data 1. [file elife-91304-fig1-figsupp4-data1.zip › Figure 1_Figure supplement 4_Source data 1_RAW membranes/F1-FS4-D_GAPDH_esiTop1.tif]

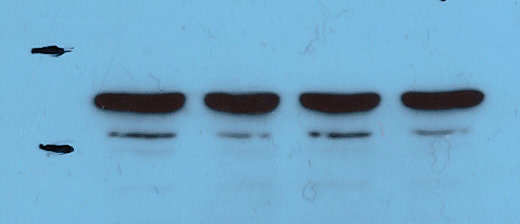

Supplement: Figure 1—figure supplement 4—source data 1. [file elife-91304-fig1-figsupp4-data1.zip › Figure 1_Figure supplement 4_Source data 1_RAW membranes/F1-FS4-D_GAPDH_esiTop2a.tif]

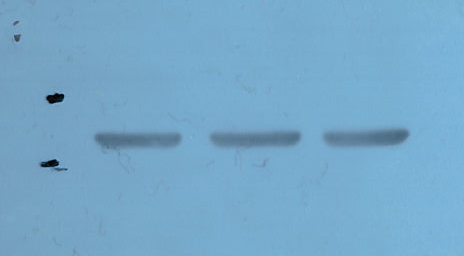

Supplement: Figure 1—figure supplement 4—source data 1. [file elife-91304-fig1-figsupp4-data1.zip › Figure 1_Figure supplement 4_Source data 1_RAW membranes/F1-FS4-D_GAPDH_RNAiTOP2b.tif]

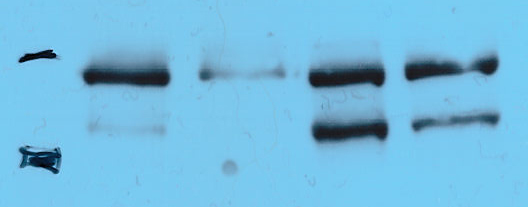

Supplement: Figure 1—figure supplement 4—source data 1. [file elife-91304-fig1-figsupp4-data1.zip › Figure 1_Figure supplement 4_Source data 1_RAW membranes/F1-FS4-D_TOP1_esiTop1.tif]

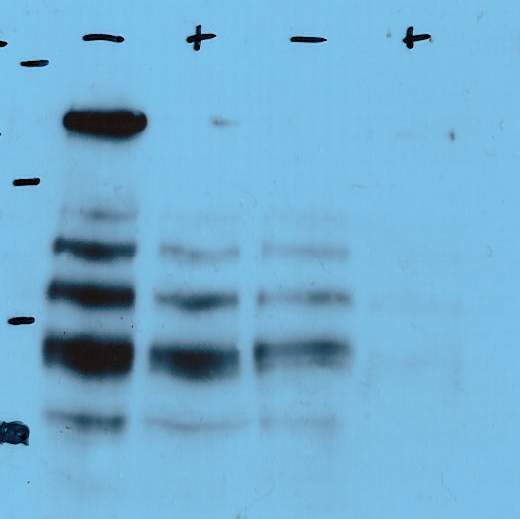

Supplement: Figure 1—figure supplement 4—source data 1. [file elife-91304-fig1-figsupp4-data1.zip › Figure 1_Figure supplement 4_Source data 1_RAW membranes/F1-FS4-D_TOP2A_esiTop2a.tif]

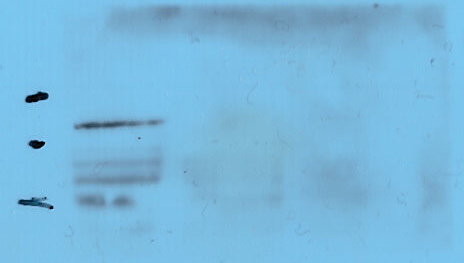

Supplement: Figure 1—figure supplement 4—source data 1. [file elife-91304-fig1-figsupp4-data1.zip › Figure 1_Figure supplement 4_Source data 1_RAW membranes/F1-FS4-D_TOP2B_RNAiTOP2b.tif]

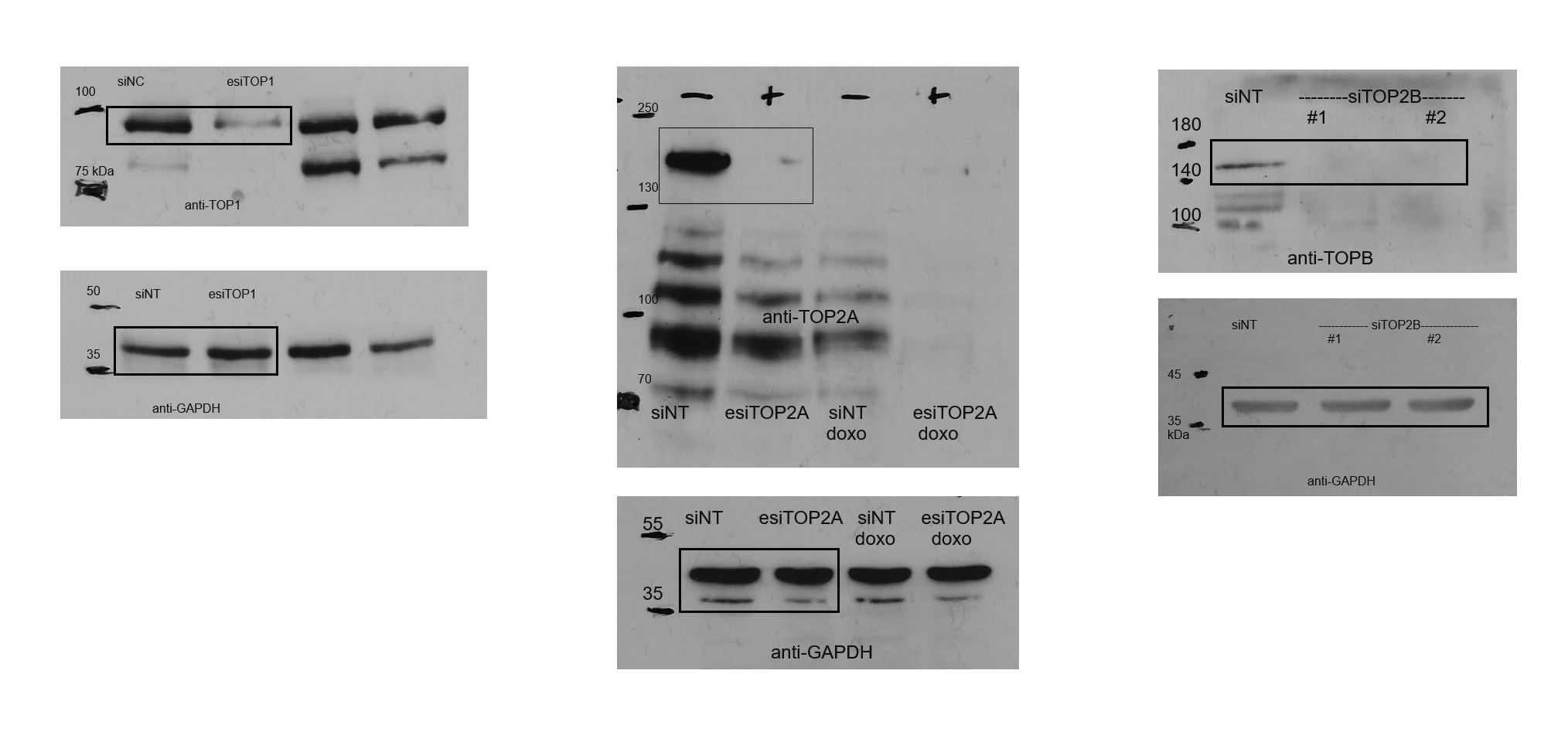

Supplement: Figure 1—figure supplement 4—source data 2. [file elife-91304-fig1-figsupp4-data2.zip › Figure 1_Figure supplement 4_Source data 2_uncropp label/Fig1_FS4-D.tif]

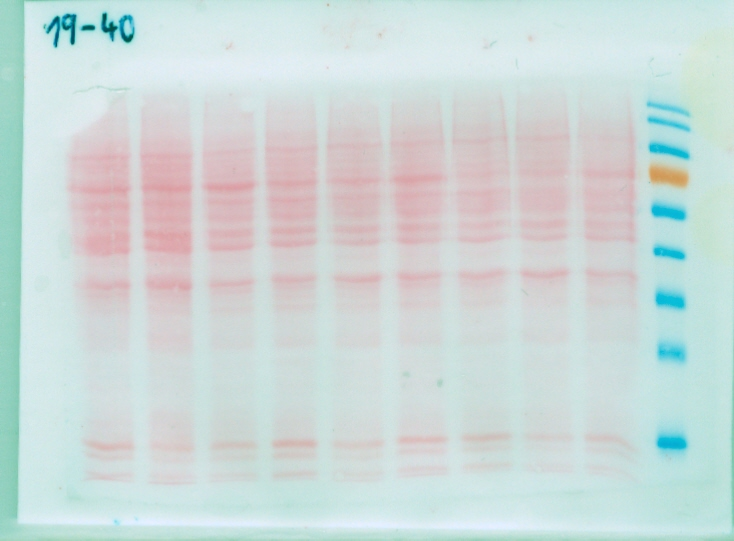

Supplement: Figure 2—figure supplement 2—source data 1. [file elife-91304-fig2-figsupp2-data1.zip › Figure 2_Figure supplement 2_Source data 1_RAW membranes/F2-FS2C_PonceauS.tif]

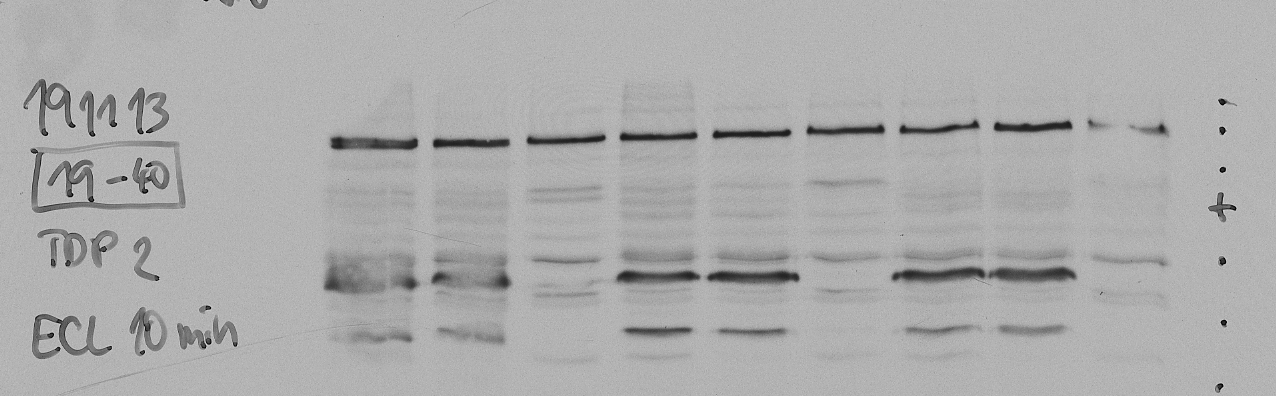

Supplement: Figure 2—figure supplement 2—source data 1. [file elife-91304-fig2-figsupp2-data1.zip › Figure 2_Figure supplement 2_Source data 1_RAW membranes/F2-FS2C_TDP2_ETP-RNAi.tif]

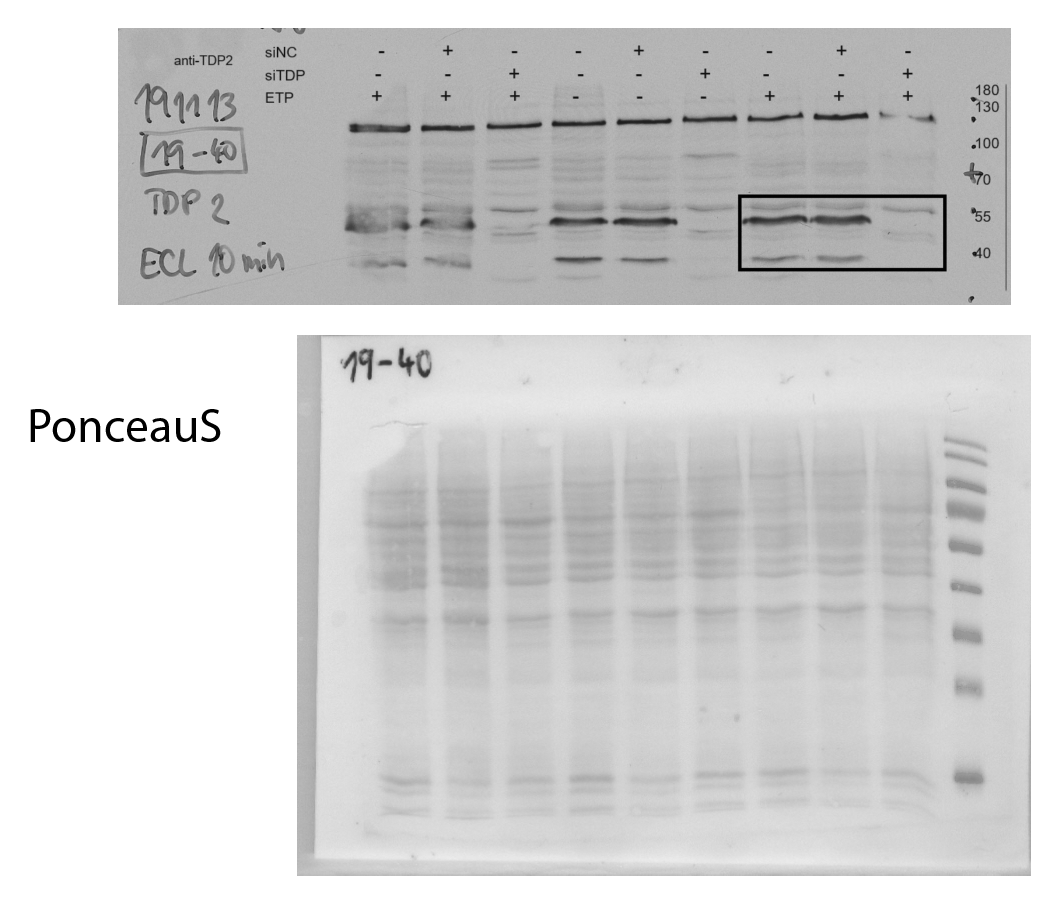

Supplement: Figure 2—figure supplement 2—source data 2. [file elife-91304-fig2-figsupp2-data2.zip › Figure 2_Figure supplement 2_Source data 2_uncropp label/F2-FS2C.tif]

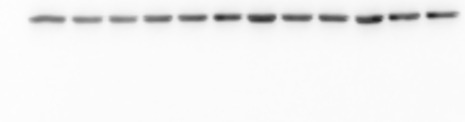

Supplement: Figure 5—figure supplement 1—source data 1. [file elife-91304-fig5-figsupp1-data1.zip › Figure 5_Figure supplement 1_Source data 1_RAW membranes/F5-FS1 A_GAPDH_ATMi.tif]

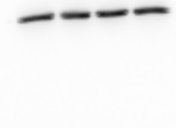

Supplement: Figure 5—figure supplement 1—source data 1. [file elife-91304-fig5-figsupp1-data1.zip › Figure 5_Figure supplement 1_Source data 1_RAW membranes/F5-FS1 A_GAPDH_ATRi.tif]

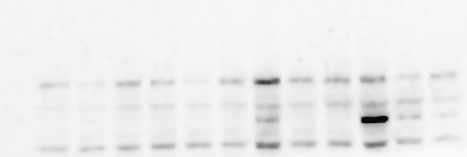

Supplement: Figure 5—figure supplement 1—source data 1. [file elife-91304-fig5-figsupp1-data1.zip › Figure 5_Figure supplement 1_Source data 1_RAW membranes/F5-FS1 A_p53_pS15_ATMi.tif]

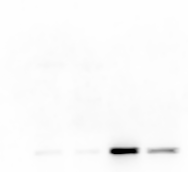

Supplement: Figure 5—figure supplement 1—source data 1. [file elife-91304-fig5-figsupp1-data1.zip › Figure 5_Figure supplement 1_Source data 1_RAW membranes/F5-FS1 A_p53_pS15_ATR.tif]

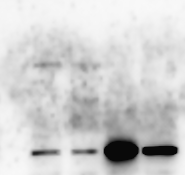

Supplement: Figure 5—figure supplement 1—source data 1. [file elife-91304-fig5-figsupp1-data1.zip › Figure 5_Figure supplement 1_Source data 1_RAW membranes/F5-FS1 A_p53_pS15_longer_ATRi.tif]

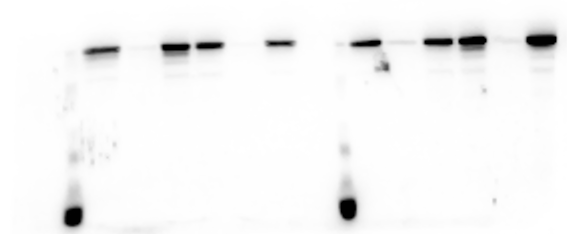

Supplement: Figure 5—figure supplement 1—source data 1. [file elife-91304-fig5-figsupp1-data1.zip › Figure 5_Figure supplement 1_Source data 1_RAW membranes/F5-FS1 D_anti-RAD51_esiRAD51.tif]

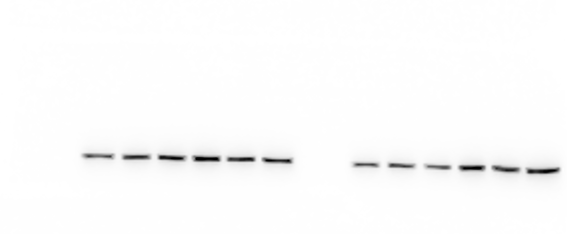

Supplement: Figure 5—figure supplement 1—source data 1. [file elife-91304-fig5-figsupp1-data1.zip › Figure 5_Figure supplement 1_Source data 1_RAW membranes/F5-FS1 D_anti-tubulin_esiRAD51.tif]

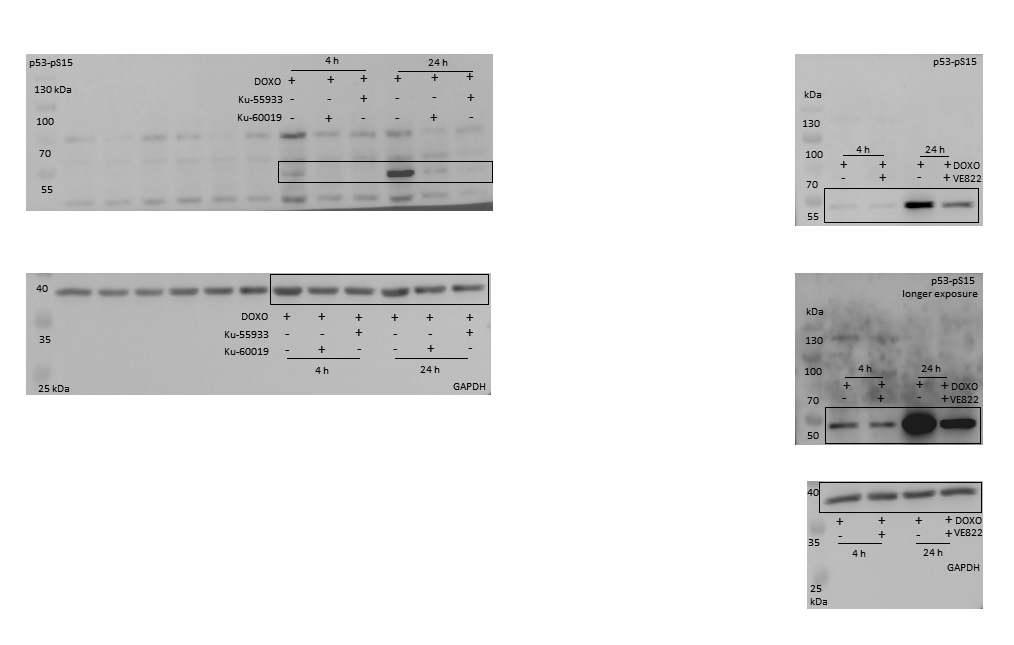

Supplement: Figure 5—figure supplement 1—source data 2. [file elife-91304-fig5-figsupp1-data2.zip › Figure 5_Figure supplement 1_Source data 2_uncropp label/F5-FS1 A.tif]

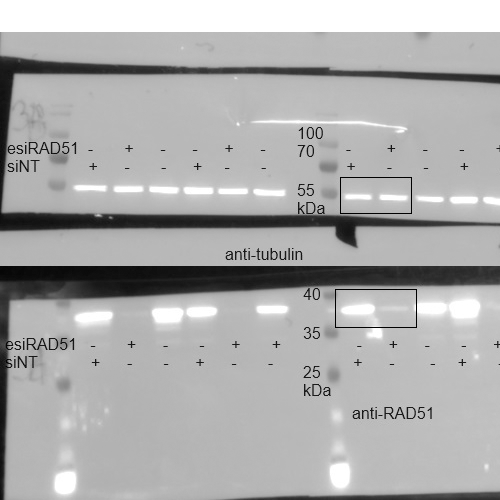

Supplement: Figure 5—figure supplement 1—source data 2. [file elife-91304-fig5-figsupp1-data2.zip › Figure 5_Figure supplement 1_Source data 2_uncropp label/F5-FS1 D.tif]
