# Supplementary material for: Topological stress triggers persistent DNA lesions in ribosomal DNA with ensuing formation of PML-nucleolar compartment
Source: eLife. 2024 Oct 10;12:RP91304. doi: 10.7554/eLife.91304 (PMC11466457; doi:10.7554/eLife.91304)
Supplement: Supplementary file 1. — The applied concentration and toxicity specification are shown. [file elife-91304-supp1.docx]

Supplementary File 1

| **Class** | **Compound** | **Concentration** | **Specification** | **Citation** |
| --- | --- | --- | --- | --- |
| **Topoisomerase inhibitors** | camptothecin | 50 µM | TOP1 poison | (1,2) |
|  | topotecan | 50 µM | TOP1 poison | (3) |
|  | doxorubicin | 0.75 µM | TOP2 poison (< 1 µM), DNA intercalation, histone eviction | (4-6) |
|  | aclarubicin | 0.05 µM | inhibition of TOP2 binding to DNA, DNA intercalation, histone eviction | (5,7) |
|  | etoposide | 50 µM | TOP2 poison | (8,9) |
| **RNAP I inhibitors** | actinomycin D | 10 nM | DNA intercalation (preferentially to GC-rich sequences) | (10,11) |
|  | BMH-21 | 0.5 µM | Intercalates DNA (preferentially to GC-rich sequences) | (12,13) |
|  | CX-5461 | 5 µM | block the release of RNAPI pre-initiation complex | (14) |
| **Inhibitors  of rRNA processing** | 5-FU | 200 µM | antimetabolite; inhibits pre-rRNA late processing | (15,16) |
|  | MG-132 | 10 µM | The proteasome inhibitor; inhibits pre-rRNA late processing | (15,17) |
|  | roscovitine | 20 µM | MAP-kinase inhibitor; inhibits pre-rRNA early processing | (15,18,19) |
| **Replicative stress inducers** | aphidicolin | 0.4 µM | DNA polymerase A, D inhibitor | (20) |
|  | hydroxyurea | 100 µM | prevent dNTP accumulation at G1/S and block DNA synthesis | (21) |
| **Others** | oxaliplatin | 10 µM | alkylating agent, DNA cross-linking, in higher concentrations inhibition of RNA and protein synthesis | (22) (23) |
|  | BrdU | 100 µM | nucleotide analogue; induces DNA damage response radio-sensitization | (24-26) |
|  | IR | 10 Gy | DNA-damaging treatment |  |
|  | IFNγ | 5 ng/mL | JAK-STAT pathway activator; induces the expression of PML | (27,28) |

1. Hsiang, Y.H., Hertzberg, R., Hecht, S. and Liu, L.F. (1985) Camptothecin induces protein-linked DNA breaks via mammalian DNA topoisomerase I. *J Biol Chem*, **260**, 14873-14878.

2. Liu, L.F., Desai, S.D., Li, T.K., Mao, Y., Sun, M. and Sim, S.P. (2000) Mechanism of action of camptothecin. *Annals of the New York Academy of Sciences*, **922**, 1-10.

3. Hsiang, Y.H. and Liu, L.F. (1988) Identification of mammalian DNA topoisomerase I as an intracellular target of the anticancer drug camptothecin. *Cancer Res*, **48**, 1722-1726.

4. Tewey, K.M., Rowe, T.C., Yang, L., Halligan, B.D. and Liu, L.F. (1984) Adriamycin-induced DNA damage mediated by mammalian DNA topoisomerase II. *Science*, **226**, 466-468.

5. Pang, B., Qiao, X., Janssen, L., Velds, A., Groothuis, T., Kerkhoven, R., Nieuwland, M., Ovaa, H., Rottenberg, S., van Tellingen, O. *et al.* (2013) Drug-induced histone eviction from open chromatin contributes to the chemotherapeutic effects of doxorubicin. *Nat Commun*, **4**, 1908.

6. Atwal, M., Swan, R.L., Rowe, C., Lee, K.C., Lee, D.C., Armstrong, L., Cowell, I.G. and Austin, C.A. (2019) Intercalating TOP2 Poisons Attenuate Topoisomerase Action at Higher Concentrations. *Molecular pharmacology*, **96**, 475-484.

7. Jensen, P.B., Sorensen, B.S., Demant, E.J., Sehested, M., Jensen, P.S., Vindelov, L. and Hansen, H.H. (1990) Antagonistic effect of aclarubicin on the cytotoxicity of etoposide and 4'-(9-acridinylamino)methanesulfon-m-anisidide in human small cell lung cancer cell lines and on topoisomerase II-mediated DNA cleavage. *Cancer Res*, **50**, 3311-3316.

8. Willmore, E., Frank, A.J., Padget, K., Tilby, M.J. and Austin, C.A. (1998) Etoposide targets topoisomerase IIalpha and IIbeta in leukemic cells: isoform-specific cleavable complexes visualized and quantified in situ by a novel immunofluorescence technique. *Molecular pharmacology*, **54**, 78-85.

9. Minocha, A. and Long, B.H. (1984) Inhibition of the DNA catenation activity of type II topoisomerase by VP16-213 and VM26. *Biochem Biophys Res Commun*, **122**, 165-170.

10. Sobell, H.M., Jain, S.C., Sakore, T.D. and Nordman, C.E. (1971) Stereochemistry of actinomycin--DNA binding. *Nat New Biol*, **231**, 200-205.

11. Chen, H., Liu, X. and Patel, D.J. (1996) DNA bending and unwinding associated with actinomycin D antibiotics bound to partially overlapping sites on DNA. *J Mol Biol*, **258**, 457-479.

12. Jacobs, R.Q., Huffines, A.K., Laiho, M. and Schneider, D.A. (2022) The small-molecule BMH-21 directly inhibits transcription elongation and DNA occupancy of RNA polymerase I in vivo and in vitro. *J Biol Chem*, **298**, 101450.

13. Peltonen, K., Colis, L., Liu, H., Jaamaa, S., Zhang, Z., Af Hallstrom, T., Moore, H.M., Sirajuddin, P. and Laiho, M. (2014) Small molecule BMH-compounds that inhibit RNA polymerase I and cause nucleolar stress. *Mol Cancer Ther*, **13**, 2537-2546.

14. Mars, J.C., Tremblay, M.G., Valere, M., Sibai, D.S., Sabourin-Felix, M., Lessard, F. and Moss, T. (2020) The chemotherapeutic agent CX-5461 irreversibly blocks RNA polymerase I initiation and promoter release to cause nucleolar disruption, DNA damage and cell inviability. *NAR Cancer*, **2**, zcaa032.

15. Burger, K., Muhl, B., Harasim, T., Rohrmoser, M., Malamoussi, A., Orban, M., Kellner, M., Gruber-Eber, A., Kremmer, E., Holzel, M. *et al.* (2010) Chemotherapeutic drugs inhibit ribosome biogenesis at various levels. *J Biol Chem*, **285**, 12416-12425.

16. Longley, D.B., Harkin, D.P. and Johnston, P.G. (2003) 5-fluorouracil: mechanisms of action and clinical strategies. *Nature reviews. Cancer*, **3**, 330-338.

17. Rock, K.L., Gramm, C., Rothstein, L., Clark, K., Stein, R., Dick, L., Hwang, D. and Goldberg, A.L. (1994) Inhibitors of the proteasome block the degradation of most cell proteins and the generation of peptides presented on MHC class I molecules. *Cell*, **78**, 761-771.

18. Vesely, J., Havlicek, L., Strnad, M., Blow, J.J., Donella-Deana, A., Pinna, L., Letham, D.S., Kato, J., Detivaud, L., Leclerc, S. *et al.* (1994) Inhibition of cyclin-dependent kinases by purine analogues. *Eur J Biochem*, **224**, 771-786.

19. Abraham, R.T., Acquarone, M., Andersen, A., Asensi, A., Belle, R., Berger, F., Bergounioux, C., Brunn, G., Buquet-Fagot, C., Fagot, D. *et al.* (1995) Cellular effects of olomoucine, an inhibitor of cyclin-dependent kinases. *Biology of the cell / under the auspices of the European Cell Biology Organization*, **83**, 105-120.

20. Wright, G.E., Hübscher, U., Khan, N.N., Focher, F. and Verri, A. (1994) Inhibitor analysis of calf thymus DNA polymerases alpha, delta and epsilon. *FEBS Lett*, **341**, 128-130.

21. Koç, A., Wheeler, L.J., Mathews, C.K. and Merrill, G.F. (2004) Hydroxyurea arrests DNA replication by a mechanism that preserves basal dNTP pools. *J Biol Chem*, **279**, 223-230.

22. Woynarowski, J.M., Chapman, W.G., Napier, C., Herzig, M.C. and Juniewicz, P. (1998) Sequence- and region-specificity of oxaliplatin adducts in naked and cellular DNA. *Molecular pharmacology*, **54**, 770-777.

23. Chaney, S.G., Campbell, S.L., Bassett, E. and Wu, Y. (2005) Recognition and processing of cisplatin- and oxaliplatin-DNA adducts. *Critical reviews in oncology/hematology*, **53**, 3-11.

24. Salic, A. and Mitchison, T.J. (2008) A chemical method for fast and sensitive detection of DNA synthesis in vivo. *Proc Natl Acad Sci U S A*, **105**, 2415-2420.

25. Kato, S., Kimura, M. and Miwa, N. (2014) Enhanced radiosensitization by the cationic liposome-encapsulated thymidine analogue BrdU through the increased intracellular BrdU-uptake on human melanoma as compared to anionic or nonionic liposomal or free BrdU. *Journal of biomedical nanotechnology*, **10**, 3280-3290.

26. Michishita, E., Nakabayashi, K., Suzuki, T., Kaul, S.C., Ogino, H., Fujii, M., Mitsui, Y. and Ayusawa, D. (1999) 5-Bromodeoxyuridine induces senescence-like phenomena in mammalian cells regardless of cell type or species. *Journal of biochemistry*, **126**, 1052-1059.

27. Briscoe, J., Rogers, N.C., Witthuhn, B.A., Watling, D., Harpur, A.G., Wilks, A.F., Stark, G.R., Ihle, J.N. and Kerr, I.M. (1996) Kinase-negative mutants of JAK1 can sustain interferon-gamma-inducible gene expression but not an antiviral state. *Embo j*, **15**, 799-809.

28. Stadler, M., Chelbi-Alix, M.K., Koken, M.H., Venturini, L., Lee, C., Saib, A., Quignon, F., Pelicano, L., Guillemin, M.C., Schindler, C. *et al.* (1995) Transcriptional induction of the PML growth suppressor gene by interferons is mediated through an ISRE and a GAS element. *Oncogene*, **11**, 2565-2573.
