# Supplementary material for: Topological stress triggers persistent DNA lesions in ribosomal DNA with ensuing formation of PML-nucleolar compartment
Source: eLife. 2024 Oct 10;12:RP91304. doi: 10.7554/eLife.91304 (PMC11466457; doi:10.7554/eLife.91304)
Supplement: Supplementary file 2. [file elife-91304-supp2.docx]

Supplementary File 2

| **Antibodies used for immunofluorescence** | | | | |
| --- | --- | --- | --- | --- |
| anti-53BP1 | mouse, monoclonal | MAB3802 | Sigma-Aldrich/Merck (Darmstadt, Germany) | 1:400 |
| anti-BrdU | mouse, monoclonal | B8434 | Sigma-Aldrich/Merck (Darmstadt, Germany) | 1:500 |
| anti-DHX9 | rabbit, polyclonal | NB110-40579 | Bio-Techne/NovusBiological (Minneapolis, USA) | 1:400 |
| anti-PML | mouse, monoclonal | sc-966 | Santa Cruz Biotechnology (Dallas, TX, USA) | 1:100 |
| anti-PML | rabbit, polyclonal | sc-5621 | Santa Cruz Biotechnology (Dallas, TX, USA) | 1:200 |
| anti-PML | rabbit, polyclonal | ABD-030 | Jena Bioscience (Jena, Germany) | 1:500 |
| anti-phosphoserine 139 of histone H2AX | mouse, monoclonal | 05-636 | Millipore/Merck (Darmstadt, Germany) | 1:500 |
| anti-phosphoserine 139 of histone H2AX | rabbit, polyclonal | 11174 | Abcam (Cambridge, UK) | 1:500 |
| anti-nucleophosmin/B23 | mouse, monoclonal | 32-5200 | Invitrogen/Thermo Fisher Scientific (Waltham, MA, USA) | 1:200 |
| anti-PAF49 | rabbit, polyclonal | ab92428 | Abcam (Cambridge, UK) | 1:250 |
| anti-Rad51 | rabbit, polyclonal |  | Gift from Pavel Janscak (University of Zurich, Switzerland) | 1:500 |
| anti-RPA194 (H-300) | rabbit, polyclonal | sc-28714 | Santa Cruz Biotechnology (Dallas, TX, USA) | 1:200 |
| anti-phospho-RPA2 (pSer33) | rabbit, polyclonal | NB100-544 | Bio-Techne/NovusBiological (Minneapolis, USA) | 1:500 |
| anti-UBTF | rabbit, polyclonal | HPA006385 | Sigma-Aldrich/Merck (Darmstadt, Germany) | 1:100 |
| Alexa Fluor 488 goat anti-mouse | goat anti-mouse | A-11029 | Invitrogene/Thermo Fisher Scientific (Waltham, MA, USA) | 1:1000 |
| Alexa Fluor 568 goat anti-mouse | goat anti-mouse | A-11031 | Invitrogene/Thermo Fisher Scientific (Waltham, MA, USA) | 1:1000 |
| Alexa Fluor 488 goat anti-rabbit | goat anti-rabbit | A-11034 | Invitrogene/Thermo Fisher Scientific (Waltham, MA, USA) | 1:1000 |
| Alexa Fluor 568 goat anti-rabbit | goat anti-rabbit | A-11036 | Invitrogene/Thermo Fisher Scientific (Waltham, MA, USA) | 1:1000 |
| **Antibodies used for immuno-FISH** | | | | |
| anti-53BP1 | mouse, monoclonal | MAB3802 | Millipore/Merck (Darmstadt, Germany) | 1:250 |
| anti-PML | Rabbit, polyclonal | sc-5621 | Santa Cruz Biotechnology (Dallas, TX, USA) | 1:400 |
| anti-nucleophosmin/B23 | mouse, monoclonal | 32-5200 | Invitrogen/Thermo Fisher Scientific (Waltham, MA, USA) | 1:300 |
| Alexa Fluor 405 goat anti-mouse | goat anti-mouse | A-31553 | Invitrogene/Thermo Fisher Scientific (Waltham, MA, USA) | 1:500 |
| Alexa Fluor 488 goat anti-rabbit | goat anti-rabbit | A-11034 | Invitrogene/Thermo Fisher Scientific (Waltham, MA, USA) | 1:500 |
| Anti-Digoxigenin-Rhodamine, Fab fragments | Sheep anti-digoxigenin | 11207750910 | Sigma-Aldrich/Merck (Darmstadt, Germany) | 1:15 |
| **Antibodies used for immunoblotting** | | | | |
| anti-GAPDH | mouse, monoclonal | GTX30666 | GeneTEX (Irvine, CA, USA) | 1:10 000 |
| anti-p53 | mouse, monoclonal | sc-126 | Santa Cruz Biotechnology (Dallas, TX, USA) | 1:500 |
| anti-p53 | mouse, monoclonal | ab1101 | Abcam (Cambridge, UK) | 1:1 000 |
| anti-topoisomerase I | rabbit, polyclonal | HPA019039 | Sigma-Aldrich/Merck (Darmstadt, Germany) | 1:1 000 |
| anti-topoisomerase I | rabbit, monoclonal | ab109374 | Abcam (Cambridge, UK) | 1:1 000 |
| anti-topoisomerase II α | mouse, monoclonal | sc-365916 | Santa Cruz Biotechnology (Dallas, TX, USA) | 1:1 000 |
| anti-topoisomerase II β | rabbit, polyclonal | sc-13059 | Santa Cruz Biotechnology (Dallas, TX, USA) | 1:1 000 |
| IgG-HRP goat anti-rabbit | goat anti-rabbit | 170-6515 | Bio-Rad (Hercules, CA, USA) | 1:10 000 |
| IgG-HRP goat anti-rabbit | goat anti-rabbit | #A6154 | Sigma-Aldrich/Merck (Darmstadt, Germany) | 1:10 000 |
| IgG-HRP goat anti-mouse | goat anti-mouse | 170-6516 | Bio-Rad (Hercules, CA, USA) | 1:10 000 |
| IgG-HRP goat anti-mouse | goat anti-mouse | #A9044 | Sigma-Aldrich/Merck (Darmstadt, Germany) | 1:10 000 |
| anti-phospho-p53 (pSer15) | mouse, monoclonal | #9286 | Cell signaling (Danvers, MA, USA) | 1:1 000 |
| anti-GAPDH | mouse, monoclonal | sc-47724 | Santa Cruz Biotechnology (Dallas, TX, USA) | 1:20 000 |
| anti-Rad51 | rabbit, polyclonal | sc-8349 | Santa Cruz Biotechnology (Dallas, TX, USA) | 1:500 |
| anti-β-tubulin | mouse, monoclonal |  | Gift from Pavel Dráber (IMG CAS, Prague; Czech Republic) | 1:10000 |
