# Supplementary material for: Topological stress triggers persistent DNA lesions in ribosomal DNA with ensuing formation of PML-nucleolar compartment
Source: eLife. 2024 Oct 10;12:RP91304. doi: 10.7554/eLife.91304 (PMC11466457; doi:10.7554/eLife.91304)
Supplement: Supplementary file 3. [file elife-91304-supp3.docx]

Supplementary File 3

| **Name of plasmid** | **Description** | **Source** |
| --- | --- | --- |
| pCDNA4TO-FKBP-PPO-HA | Plasmid with a regulable expression of I-PpoI | (1); a kind gift of Libor Macurek (IMG CAS, Prague; Czech Republic) |
| pLVX-TET-ONE-puro | All-in-one lentiviral vector with puromycin resistance and strong inducible expression of genes using the Tet-On® system | Clontech Laboratories, Inc.; A Takara Bio Company |
| pCDH-CMV-MCS-EF1-Neo | Cloning and Expression Lentivector | System Biosciences, LLC (Palo Alto, CA 94303) |
| pLVX-TET-ONE-neo | All-in-one lentiviral vector with neomycin resistance and strong inducible expression of genes using the Tet-On® system. | This work |
| pLVX-TET-One-neo-FKBP-PPO-HA | lentiviral vector with neomycin resistance enabling the preparation of stable cell lines with a regulable expression of I-PpoI. | This work |
| pUC-hrDNA-12.0 | Plasmid bearing DNA of rDNA intergenic spacer. The source for the FISH probe targeting rDNA locus. | (2); a kind gift of Prof. Brian McStay (NUI Galway, Ireland) |
| BAC_CH507-535F5 | BAC bearing DNA of human distal junction. The source for the FISH probe targeting DJ locus. | BACPAC Resources Center (Emeryville, CA, USA) |

**1.** Warmerdam, D.O., van den Berg, J. and Medema, R.H. (2016) Breaks in the 45S rDNA Lead to Recombination-Mediated Loss of Repeats. *Cell reports*, 14, 2519-2527.

2. van Sluis, M., van Vuuren, C., Mangan, H. and McStay, B. (2020) NORs on human acrocentric chromosome p-arms are active by default and can associate with nucleoli independently of rDNA. *Proc Natl Acad Sci U S A*, 117, 10368-10377.
