# Supplementary material for: Topological stress triggers persistent DNA lesions in ribosomal DNA with ensuing formation of PML-nucleolar compartment
Source: eLife. 2024 Oct 10;12:RP91304. doi: 10.7554/eLife.91304 (PMC11466457; doi:10.7554/eLife.91304)
Supplement: Supplementary file 4. [file elife-91304-supp4.docx]

| Name of Primer | **Application** | **Sequence** |
| --- | --- | --- |
| GA-PpoI-LVXpur-fr-F | cloning | ACTTCCTACCCTCGTAAAGaagcttggtaccgagctcg |
| GA-PpoI-LVXpur-fr-R | cloning | GCAGGGGAGGTGGTCTGGATCCccatagagcccaccgcat |
| GA-V-LVXpuro-F | cloning | GTTCTTCTGAACGCGTCTGGAACAATCA |
| GA-V-LVXpuro-R | cloning | GTTCAATCATGGTCGCGTTTTGCAAAAG |
| GA-F-neo-R | cloning | CCAGACGCGTTCAGAAGAACTCGTCAAGAAGGCGATAGAAG |
| GA-F-neo-F | cloning | AAACGCGACCATGATTGAACAAGATGGATTGCAC |
| LIG4_F | qPCR | GAAGGCATCTGGTAAGCTCG |
| LIG4_R | qPCR | AGCTTCCTTAGCACTCACACA |
| GAPDH fw | qPCR | GTCGGAGTCAACGGATTTGG |
| GAPDH rev | qPCR | AAAAGCAGCCCTGGTGACC |

**Supplementary File 4**
